# Morphological and acrosomal changes of canine spermatozoa during epididymal transit

**DOI:** 10.1186/1751-0147-55-17

**Published:** 2013-02-26

**Authors:** Sara Varesi, Valentina Vernocchi, Massimo Faustini, Gaia Cecilia Luvoni

**Affiliations:** 1 Dipartimento di Scienze Veterinarie per la Salute, la Produzione Animale e la Sicurezza Alimentare, Università degli Studi di Milano, Via Celoria 10, 20133, Milan, Italy; 2 Dipartimento di Scienze Veterinarie e Sanità Pubblica, Università degli Studi di Milano, Via Celoria 10, 20133, Milan, Italy

**Keywords:** Dog, Spermatozoa, Epididymis

## Abstract

**Background:**

During epididymal transit, functional and structural modifications leading to full maturation enable male gametes to reach, recognize and fertilize the oocytes. In dogs, little is known on the modifications of spermatozoa during the passage in the epididymis. The aim of this study was to describe the motility, morphology and acrosomal patterns of canine spermatozoa retrieved from the epididymis caput, corpus and cauda.

**Results:**

After the dilution required for the collection of epididymal content, sperm motility was significantly higher (*P* <0.0001) in the cauda compared to corpus and caput.

Proportions of spermatozoa with normal morphology were significantly higher in corpus (*P* =0.02) and cauda (*P* <0.0001) compared to caput. Overall morphological abnormalities of the head and neck/midpiece were similar in the three different epididymal regions. A significantly increased prevalence of tail defects, mainly represented by single bent tails, was observed in the corpus compared to caput (*P* <0.0001) and cauda (*P* =0.006).

Numbers of immature sperm with cytoplasmic droplets decreased from the proximal to the distal region of the epididymis. Particularly, proximal cytoplasmic droplets were more frequently found in spermatozoa collected from the caput epididymis than in the corpus (*P* <0.0001) and in the cauda (*P* <0.0001), whereas the occurrence of distal cytoplasmic droplets was higher in the corpus than in the caput (*P* =0.0003) and in the cauda (*P* <0.05).

Significantly higher proportions of spermatozoa with intact acrosomes were retrieved from the cauda epididymis than from the caput (*P* =0.03) and the corpus (*P* =0.008). This difference was mainly due to a lower proportion of spermatozoa with abnormal acrosomes (mainly swollen acrosomes) rather than with absent acrosomes.

**Conclusions:**

Canine spermatozoa undergo several modifications in the epididymis. The acquisition of progressive motility, migration of the cytoplasmic droplet and acrosomal reshaping lead to mature spermatozoa which are then stored in the cauda epididymis. From this site, spermatozoa can be retrieved and used in assisted reproductive techniques as a valuable tool for propagating genetic traits of high value individuals that dies accidentally or undergoes orchiectomy for medical purposes. Further investigations should be also focused on the potential use of spermatozoa recovered from other epididymal regions.

## Background

In the mammalian epididymis, substantial changes of spermatozoa occur. During epididymal transit from caput to cauda, functional and structural modifications leading to full maturation enable male gametes to reach, recognize and fertilize the oocytes.

Maturational changes of spermatozoa have been described in different species including humans. Gradual modifications in motility and morphology have been observed in spermatozoa collected from different regions of the epididymis [1-7].

Previous studies proposed a further role of the epididymis in the recognition and removal of abnormal spermatozoa [4,8,9]. In addition, some authors hypothesized that the epididymis might be a site where sperm abnormalities develop [2,3,10,11].

In dogs, little is known on the post-testicular modifications of spermatozoa during the passage of the epididymis, whereas their fertilizing ability has been demonstrated by birth of offspring following artificial insemination with gametes retrieved from the cauda epididymis [12-16].

Examination of canine spermatozoa obtained from different regions of the epididymis has been done only in one study [13]. In that study, the organ was divided only in two portions (caput/corpus and cauda) and the samples collected from the caput and the corpus were not differentiated. Furthermore, a detailed description of site and type of morphological abnormalities and of acrosomal patterns were not reported.

Additional information on the morphological and acrosomal changes of epididymal spermatozoa would contribute to clarify some aspects of the maturational process and of the potential above mentioned roles of the epididymis.

 The aim of this study was to describe the characteristics of spermatozoa retrieved from the different regions of the canine epididymis. For this purpose, motility, morphology and acrosomal patterns of spermatozoa obtained from caput, corpus and cauda epididymis were compared.

## Methods

All chemicals were purchased from the Sigma Chemical Company (St. Louis, MO, USA) unless otherwise stated.

### Animals

Thirteen healthy and pubertal privately owned stud dogs, aged between 1 and 2.5 years (8 to 33 kg body weight), presented to the Department for routine orchiectomy were included in this study.

### Epididymal spermatozoa retrieval

Thirteen pairs of canine gonads were transported to the laboratory within 10 min after surgical removal. The epididymis was dissected from each testis and pampiniform plexus using a scalpel blade. The small vessels were removed with scissors to reduce contamination by blood, and then each epididymis was macroscopically divided into three portions, caput, corpus and cauda, according to Schimming et al. [17,18].

Each portion was placed in a Petri dish containing 4 ml of Ham’s F-10 medium supplemented with 2 mmol glutamine, 100 IU/ml Na-benzyl penicillin, 0.1 mg/ml streptomycin sulphate, and 5% fetal bovine serum (mOsm 285). The different tracts were minced with a scalpel blade, and after 30 min of incubation at 37°C, 1 ml of suspension was collected from each dish and processed for spermatozoa evaluation.

### Spermatozoa evaluation

Sperm concentration was determined with a Bürker chamber. After the dilution required for the collection of epididymal content, motility was subjectively assessed by the same investigator with a light microscope (40x) with a heated stage at 38°C. Spermatozoa were con sidered to be motile only if they exhibited progressive motility of a score of at least 3 or 4 on a scale of 0–4 (0, absent; 1, weak or sluggish; 2, definite; 3, good; 4, vigorous) [19].

Morphology of spermatozoa was assessed following staining of the smear with Bengal Rose and Victoria Blue B. A total of 100 spermatozoa was evaluated under light microscope with oil immersion objective at 100x magnification. Normal spermatozoa and site of defects in abnormal spermatozoa (head, neck/midpiece, tail) were recorded [20]. For each abnormal sperm all the anomalies of different sites were considered [21]. Abnormal sperm heads included those that were pear-shaped, large, small, or amorphous. Alterations of the neck/midpiece included bent neck, bent and thick midpiece; abnormal tail included single bent and coiled tail. Immature sperm with proximal and distal cytoplasmic droplet were recorded separately.

The acrosome integrity was evaluated by staining spermatozoa with Peanut agglutinin (PNA) conjugated with fluorescein isothiocyanate (FITC) and propidium iodide (PI) according to the procedure described previously for stallion spermatozoa [22]. Staining solution was prepared with 90 μl of FITC-PNA (40 μg/ml in Phosphate Buffered Saline - PBS) added with 10 μl of PI (340 μM in PBS).

An amount of 10 μl of sperm suspension was smeared on a microscope slide and fixed in 96% ethanol for 30 seconds. The slide was dried in dark, and then a droplet of 20 μl of FITC-PNA/PI was added to the slide. The slide was incubated in a moist chamber at 4°C and after 30 min it was rinsed with 4°C distilled water and air dried at 4°C in dark overnight. At least 100 spermatozoa were evaluated under fluorescent microscope (Axiovert 100, Zeiss, Germany). The intact acrosome was stained green, whereas the head of the sperm was stained red.

The observed fluorescence images of ethanol-permeabilized spermatozoa, stained with FITC-PNA/PI, were classified into three patterns: 1) spermatozoa displaying intensively bright fluorescence of the acrosomal cap as “intact acrosome”; 2) spermatozoa displaying disrupted, patch-like, fluorescence of the acrosomal cap or swollen acrosomal cap as “abnormal acrosome”; 3) spermatozoa displaying a fluorescent band at the equatorial segment or displaying no fluorescence as “absent acrosome”.

### Statistical analysis

Data were resumed as mean ± standard deviation. Mean concentration, motility, normal morphology, type of abnormalities, acrosomal patterns were analyzed by a mixed linear model by a GLM procedure, taking into account the region as fixed factor and the subject as random factor, in order to reduce the error variability due to the animal. The overall morphological abnormalities on spermatozoa site (head, neck/midpiece, tail) and the overall immature spermatozoa were analyzed by one-way ANOVA, followed by the Tukey-Kramer test for multiple comparisons. *P* -values <0.05 were considered to be significant. All statistical procedures were performed by the software SAS release 9.13 for Windows platform.

The variables motility, site of abnormalities (head, neck/midpiece, tail), cytoplasmic droplets (proximal and distal) and acrosomal patterns (abnormal and absent acrosome) were processed by principal component analysis (PCA) in order to evaluate the behavior of these variables in the multivariate space.

## Results

Sperm concentration (spz × 10^6^/ml) was significantly higher (*P* =0.002) in the samples collected from the cauda (138.1 ± 161.5) compared to those collected from the caput (11.4 ± 16.7), while no differences were observed among corpus (61.4 ± 43.7) and the other regions.

Sperm motility (%) after dilution increased progressively in samples collected from caput to cauda. Proportions of motile cells were significantly higher in the distal region (53.1 ± 25.9) compared to corpus (16.2 ± 11.6; *P* <0.0001) and caput (1.3 ± 2.1; *P* <0.0001). In the caput most of the cells that did not progress showed a flagellating tail.

Morphology of spermatozoa retrieved from different epididymal regions are summarized in Table 1.

**Table 1:** Morphology of canine spermatozoa retrieved from different epididymal regions

**Spermatozoa**			**Caput**	**Corpus**	**Cauda**
Normal			24.7 ± 11.9^a^	39.0 ± 13.8^b^	50.5 ± 13.3^b^
Abnormal	HEAD	pear-shaped	2.0 ± 6.9	3.2 ± 9.6	3.6 ± 12.7
		small	0.2 ± 0.6	0.2 ± 0.6	0.2 ± 0.6
		large	0.1 ± 0.3	0	0
		amorphous	0.2 ± 0.4	0.2 ± 0.4	0
		*Total abnormalities*	2.5 ± 6.8	3.5 ± 9.5	3.8 ± 12.7
	NECK/MIDPIECE	bent neck	4.8 ± 4.0	4.7 ± 2.5	6.2 ± 3.5
		bent midpiece	1.9 ± 1.5	1.4 ± 2.1	2.2 ± 1.7
		thick midpiece	0.4 ± 0.8	0.2 ± 0.6	0.2 ± 0.4
		*Total abnormalities*	7.1 ± 5.0	6.3 ± 4.0	8.5 ± 4.7
	TAIL	single bent	15.1 ± 7.5^a^	33.8 ± 11.8^b^	21.6 ± 8.6^a^
		coiled	11.0 ± 11.0	6.2 ± 5.9	7.4 ± 7.3
		*Total abnormalities*	26.2 ± 12.5^a^	40.1 ± 11.1^b^	29.0 ± 13.6^a^
Immature	CYTOPLASMIC DROPLET	proximal	44.8 ± 19.5^a^	8.9 ±6.8^b^	3.6 ± 3.9^b^
		distal	4.7 ± 6.8^a^	20.0 ± 10.4^b^	11.4 ± 9.0^a^
		*Total immature*	49.6 ± 20.1^a^	28.9 ± 11.9^b^	15.0 ± 11.0^c^

Data are percentages, presented as mean ± SD.

Different superscripts within rows (abc) indicate significant differences (*P*<0.05).

Proportions of spermatozoa with normal morphology were significantly higher in corpus (*P* =0.02) and cauda (*P* <0.0001) compared to caput. Overall morphological abnormalities of the head and neck/midpiece were similar in the three different epididymal regions. A significantly increased prevalence of tail defects, mainly represented by single bent tails, was observed in the corpus compared to caput (*P* <0.0001) and cauda (*P* =0.006).

Numbers of immature sperm with cytoplasmic droplets decreased from the proximal to the distal region of the epididymis. Particularly, proximal cytoplasmic droplets were more frequently found in spermatozoa collected from the caput epididymis than in the corpus (*P* <0.0001) and in the cauda (*P* P<0.0001), whereas the occurrence of distal cytoplasmic droplets was higher in the corpus than in the caput (*P* =0.0003) and in the cauda (*P* <0.05).

Significantly higher proportions of spermatozoa with intact acrosomes were retrieved from the cauda epididymis than from the caput (*P* =0.03) and the corpus (*P* =0.008). This difference was mainly due to a lower proportion of spermatozoa with abnormal acrosomes (mainly swollen acrosomes) rather than with absent acrosomes (Table 2).

**Table 2:** Acrosomal patterns of canine spermatozoa retrieved from different epididymal regions

**Acrosome patterns**	**Caput**	**Corpus**	**Cauda**
Intact (%)	35.4 ± 22.4a	31.6 ± 17.4a	49.5 ± 19.9b
Abnormal (%)	61.7 ± 22.2a	62.1± 16.7a	41.5 ± 18.9b
Absent (%)	2.9 ± 2.6a	6.3± 4.0b	9.0± 3.2c

Data are percentages, presented as mean ± SD.

Different superscripts within rows (abc) indicate significant differences (*P*<0.05).

**Figure 1:**
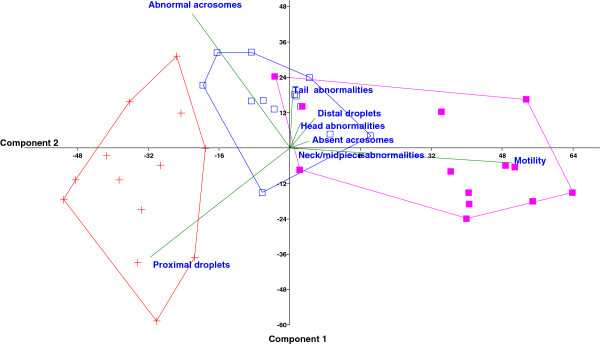
**Principal component biplot of variables in the space of the first two principal components (PC).** The length of each vector represents the weight of the variable in the plane, and the angle between variables quantifies the correlations among the variables. The objects (i.e. the samples) dispose themselves in the space of the PC relating to the variables: an object close to the end of a vector is highly correlated to the variable. Convex hulls include the spermatozoa samples from caput (cross), corpus (hollow square), and cauda (full square).

The results for PCA analysis are reported in Figure 1.

The space of the first two principal components shows that motility, abnormal acrosomes, and proximal cytoplasmic droplets are the most representative variables, and that the multivariate characteristics of the three regions are quite distinct from each other. Motility is negatively correlated with abnormal acrosomes and proximal cytoplasmic droplet in the multivariate space (univariate correlations: motility-abnormal acrosomes, r=−0.37, *P* <0.05; motility-proximal cytoplasmic droplet, r=−0.51, *P* <0.01).

## Discussion

This study describes the characteristics of canine spermatozoa collected from the caput, corpus and cauda epididymis with the aim of highlighting the modifications occurring during transit.

It is well known that some modifications of mammalian spermatozoa occurring in the epididymis are related to the maturational process that involves functional and structural changes of the gametes. Among functional changes, the capacity for sperm motility is gradually acquired from caput to cauda with a quantitative and qualitative modification of its patterns from only a faint twitch of the flagellum to a progressive and vigorous forward movement [4,23].

In the present study, canine spermatozoa collected from the cauda of the epididymis showed a higher motility compared to those retrieved from caput and corpus in addition to a high concentration due to the storage role of this area. Spermatozoa in the caput often displayed a flagellating movement of the tail instead of being immotile, as also observed in cats [3].

Concomitant with these functional changes, spermatozoa undergo structural modifications during epididymal transit such as migration of the cytoplasmic droplet and acrosomal reshaping in order to achieve the normal morphology of mature spermatozoa [4].

Cytoplasmic droplets develop during normal spermatogenesis and represent a residue of the cytoplasm after Sertoli cells have phagocytized most spermatidic cytoplasm [6]. The migration from the proximal to the distal end of the midpiece takes place in a specific region of the epididymis, which varies slightly among species. In cats, the migration occurs in the terminal part of the corpus [3], whereas in donkeys occurs from the first to the second half of the corpus [7]. In other species such as rabbit, bull and boar, this change occurs earlier either in the caput or in the passage from caput to corpus [5,24,25].

In dogs, the present results showed that the highest proportion of spermatozoa with proximal cytoplasmic droplets was in the caput. In the corpus, an abrupt decrease in frequency of proximal cytoplasmic droplets concomitant with a significant increase in the number of spermatozoa showing the distal droplet, suggests that this region is the site of migration in this species.

Concerning acrosomal reshaping, it has been demonstrated in the rabbit that the acrosome dimensions of spermatozoa collected from the caput are greater than those of spermatozoa from the cauda. During epididymal passage, these swollen acrosomes contract and localize adjacent to the nuclear surface of the sperm head [1]. In the present work, the occurrence of abnormal acrosomes, mainly represented by swollen acrosomes, was higher in the caput and in the corpus compared to the cauda where a higher proportion of spermatozoa had normal acrosomes. This gradual change toward a normal shape of the acrosome might be due to the reshaping during maturation from the proximal to the distal epididymal region as observed in the rabbit [1].

However, Axnér and co-workers [3] suggested that the decrease of feline spermatozoa with abnormal acrosomes in the cauda might also be due to the epididymal recognition of these spermatozoa as being abnormal. Besides the well-known function of the epididymis in sperm maturation, an additional role in “sperm quality control” through the removal of abnormal spermatozoa by different mechanisms (i.e. phagocytosis, dissolution by ubiquitination and degradation by other proteins) has been proposed [8,9]. However, the elimination of abnormal spermatozoa in the reproductive tract is still controversial [4].

In the present study, the proportion of canine spermatozoa with normal morphology increased significantly from caput to cauda epididymis. This increase was mainly due to the reduction of immature spermatozoa. It remains to be elucidated whether the decrease of immature spermatozoa is due to the effective maturation or to other mechanisms for removal of abnormal gametes. Frequency of anomalies of the head, neck/midpiece and tail did not differ between caput and cauda. In the cat, a significant decrease of the spermatozoa with abnormalities of testicular origin (i.e. head defects) has been described from the efferent ducts to the cauda [3]. A decreased frequency of anomalies of the midpiece, including cytoplasmic droplets, was observed among testicular and epididymal spermatozoa in the rabbit, whereas the comparison between caput and cauda epididymis failed to detect significant decrease in the frequencies of all the defects [25], as observed in this study.

On the other hand, the epididymis has been considered a site where some peculiar sperm anomalies develop [2,3,10,11]. A significant increase in sperm tail abnormalities between the proximal to the distal regions of the epididymis was reported in the cat [3]. In the boar, some types of sperm malformations of the tail were observed more frequently in the cauda, whereas other sperm defects were more uniformly distributed along the epididymis [2].

Although the proportions of abnormal canine spermatozoa between caput and cauda did not differ, the frequency of single bent tail in the corpus was significantly higher than in the other epididymal compartments, and it was often associated with presence of a distal droplet. In some cases, the distal droplet was localized along the flagellum, rather than at the distal end of the midpiece, and the tail was bent on the droplet. A possible explanation of this association is the premature release of hydrolytic enzymes by the droplet. This might produce digestion and disorganization of structural components of the tail with consequent weakness of the structure and folding of the flagellum, as hypothesized for boar spermatozoa [2]. The single bent tail may indeed be considered as an abnormality originating in the epididymis, but as it correlates with the presence of the droplet, it is also linked to the maturational process.

The analysis of the variables in a multivariate space underlines that the traits of immaturity (i.e. low motility, proximal cytoplasmic droplet, and abnormal acrosomes) show the greatest variability in the epididymal spermatozoa, confirming that the epididymis has a crucial role in sperm maturation also in dogs as previously reported in other species.

Epididymal spermatozoa represent an important source of germplasm. It would be interesting to evaluate whether the immaturity traits of spermatozoa (i.e. cytoplasmic droplet or swollen acrosome) recovered from different compartments negatively influence the fertilization.

It has been reported that ejaculated spermatozoa with proximal droplet have poor adherence to the zona pellucida in different mammalian species [6] including dogs [26]. However, the presence of the proximal droplet in the ejaculated spermatozoa is a sign of a defective sperm maturation process that could be associated with biochemical alterations interfering with the normal progress of capacitation [26], whereas in the epididymis the presence of the droplet represents a physiological condition of the gametes. For this reason, an in-depth study of fertilizing ability of epididymal spermatozoa retrieved from the entire organ would contribute to extend their use in assisted reproductive techniques.

## Conclusions

Canine spermatozoa undergo several modifications in the epididymal environment. The acquisition of progressive motility, migration of cytoplasmic droplet and acrosomal reshaping lead to mature spermatozoa which are then stored in the cauda epididymis. From this site, spermatozoa can be retrieved and used in assisted reproductive techniques as a valuable tool for propagating genetic traits of high value individuals that dies accidentally or undergoes orchiectomy for medical purposes. Further investigations should focus on the potential use of spermatozoa recovered from other epididymal regions.

## Competing interest

None of the authors have any conflict of interest to declare.

## Authors’ contributions

GCL and SV contributed to design the study, analysed the data and drafted the paper. Laboratory work was carried out by SV and VV. MF performed statistical analysis. All authors read and approved the final version of the manuscript.
